# HSD11B1 rs846908 polymorphisms and tacrolimus concentrations: quantum chemical analysis and implication in patients with renal transplantation

**Published:** 2016-01-18

**Authors:** Beuy Joob, Viroj Wiwanitkit

**Affiliations:** ^1^Sanitation 1 Medical Academic Center, Bangkok, Thailand; ^2^Visiting Professor, Hainan Medical University, Haikou, China ; Visiting Professor, Faculty of Medicine, University of Nis, Serbia; adjunct professor, Joseph Ayobabalola University, Nigeria; Honorary Professor, Dr DY Patil Medical University, India

**Keywords:** Tacrolimus, Transplantation, Polymorphism, Kidney

**Implication for health policy/practice/research/medical education:**

Tacrolimus is a widely used immunosuppressive drug for post renal transplantation. The concentration of tacrolimus is important for success in management of renal recipients. The effect of genetic polymorphism on the concentration of tacrolimus is very interesting. Here, the author studied the SD11B1 rs846908 polymorphisms and tacrolimus concentrations by quantum chemical analysis. It can be seen that the AA + AA polymorphism results in less finalized tacrolimus concentration comparing to GG + GA polymorphism.

## Introduction

Renal transplantation is the therapeutic of choice for the end stage renal disease. With advanced immunosuppressive drug, the outcome of renal transplantation becomes favorable in the present day (1). Tacrolimus is a widely used immunosuppressive drug for post-renal transplantation (2). The concentration of tacrolimus is important for success in management of the patient. The effect of genetic polymorphism on the concentration of tacrolimus is very interesting (3). Of several genes, the 11beta-hydroxysteroid dehydrogenase type 1 (HSD11B1) is widely discussed at present (4). Here, the author studied the HSD11B1 rs846908 polymorphisms and tacrolimus concentrations by quantum chemical analysis. It can be seen that the AA + AA polymorphism results in less finalized tacrolimus concentration comparing to GG + GA polymorphism.

## Materials and Methods

This work is designed as a quantum chemistry analysis using mathematical modeling study. The basic consideration is HSD11B1 gene is the origin for further proteinogenesis and subsequence further biological process and expression. The final outcome is the tacrolimus concentration. The assumption is there is a direct linkage, without any intermediate interruption, from HSD11B1 gene to tacrolimus concentration. The amount of HSD11B1 gene is therefore the determinant of tacrolimus concentration. This process can be seen in any genetic polymorphism of HSD11B1. To calculate, the final concentration of tacrolimus in mutation, standard quantum chemical analysis is used. This is the same concept as published in previous publications (5,6). As a primary assumption, in wild type, the outcome concentration of tacrolimus is firstly assigned to be “X” mol per gram of original HSB11B1 rs846908. The variability in this modeling study is the change in substrate, due to polymorphism. The studied polymorphisms are AA + AA and GG + GA.

## Results

The results from this study are shown in Table 1. It can be seen that the polymorphism GG + GA has a higher amount per mol than AA + AA. Therefore, predicted outcome concentration of tacrolimus is higher in polymorphism GG + GA. The predicted concentration of tacrolimus in AA + AA is 1.0889 time more than that of GG + GA.

**Table 1:** Predicted outcome concentration of tacrolimus

**Types**	**Amount per mol of HSD11B1 rs846908 (g)**	**predicted outcome concentration of tacrolimus (mol)**
AA + AA	540.5068	540.5068X
GG + GA	588.5050	588.5050X

## Discussion

Effect of HSD11B1 gene polymorphism is mentioned in several clinical problems in medicine (4,7,8), moreover its effect on renal transplantation and the concentration of tacrolimus is also mentioned. Recently, a model study could reveal the importance of glucocorticoid and fat in determining dosage requirement of tacrolimus in post renal transplantation status (9). Hence, the role of HSD11B1 gene polymorphism is very interesting. Here, the authors assess the interrelationship between HSD11B1 rs846908 polymorphisms and tacrolimus concentration.

In this study, it can be found that GG + GA polymorphism result in more amount or higher concentration of tacrolimus than AA + AA polymorphism. This is concordant with the recent clinical finding by Liu et al (4). Nevertheless, in the report by Liu et al, the concentration of tacrolimus in case of GG + GG polymorphism is about 1.3648 times higher than that of AA + AA polymorphism. This can confirm that, other factors like, other genetic polymorphisms, epigenetic factors, other concurrent diseases, underlying physiology and pathology of the kidney, still existed to determine the outcome of tacrolimus concentration.

## Conclusion

Tacrolimus is a widely used immunosuppressive drug for post renal transplantation. The concentration of tacrolimus is important for success in management of renal recipients. The effect of genetic polymorphism on the concentration of tacrolimus is very interesting. Here, the author studied the SD11B1 rs846908 polymorphisms and tacrolimus concentrations by quantum chemical analysis. It can be seen that the AA + AA polymorphism results in less finalized tacrolimus concentration comparing to GG + GA polymorphism.

## Authors’ contribution

VW and BJ wrote the manuscript equally.

## Conflicts of interest

The author declared no competing interests.

## Ethical considerations

Ethical issues (including plagiarism, data fabrication, double publication) have been completely observed by authors.

## Funding/Support

None.
